# Comparative evaluation of (1, 3)-β-D-glucan, mannan and anti-mannan antibodies, and *Candida *species-specific snPCR in patients with candidemia

**DOI:** 10.1186/1471-2334-7-103

**Published:** 2007-09-04

**Authors:** Fasahat F Alam, Abu S Mustafa, Zia U Khan

**Affiliations:** 1 Department of Microbiology, Faculty of Medicine, Kuwait University, Safat 13110, P. O. Box 24923, Kuwait

## Abstract

**Background:**

Candidemia is a major infectious complication of seriously immunocompromised patients. In the absence of specific signs and symptoms, there is a need to evolve an appropriate diagnostic approach. A number of methods based on the detection of *Candida *mannan, nucleic acid and (1,3)-beta- D- glucan (BDG) have been used with varying specificities and sensitivities. In this retrospective study, attention has been focused to evaluate the usefulness of two or more disease markers in the diagnosis of candidemia.

**Methods:**

Diagnostic usefulness of Platelia *Candida *Ag for the detection of mannan, Platelia *Candida *Ab for the detection of anti-mannan antibodies, Fungitell for the detection of BDG, and of a semi-nested PCR (snPCR) for the detection *Candida *species-specific DNA have been retrospectively evaluated using 32 sera from 27 patients with culture-proven candidemia, 51 sera from 39 patients with clinically suspected candidemia, sera of 10 women with *C. albicans *vaginitis, and sera of 16 healthy controls.

**Results:**

Using cut-off values recommended by the manufacturers, the sensitivity of the assays for candidemia patients were as follows: *Candida *snPCR 88%, BDG 47%, mannan 41%, anti-mannan antibodies 47%, respectively. snPCR detected 5 patients who had candidemia due to more than one *Candida *species. The sensitivities of the combined tests were as follows: *Candida *mannan and anti-mannan antibodies 75%, and *Candida *mannan and BDG 56%. Addition of snPCR data improved the sensitivity further to 88%, thus adding 10 sera that were negative by BDG and/or mannan. In clinically suspected, blood culture negative patients; the positivities of the tests were as follows: *Candida *DNA was positive in 53%, BDG in 29%, mannan in 16%, and anti-mannan antibodies in 29%. The combined detection of mannan and BDG, and mannan, BDG and *Candida *DNA enhanced the positivity to 36% and 54%, respectively. None of the sera from *Candida *vaginitis patients and healthy subjects were positive for *Candida *DNA and mannan.

**Conclusion:**

The observations made in this study reinforce the diagnostic value of snPCR in the sensitive and specific diagnosis of candidemia and detection of more than one *Candida *species in a given patient. Additionally, in the absence of a positive blood culture, snPCR detected *Candida *DNA in sera of more than half of the clinically suspected patients. While detection of BDG, mannan and anti-mannan antibodies singly or in combination could help enhancing sensitivity and eliminating false positive tests, a more extensive evaluation of these assays in sequentially collected serum samples is required to assess their value in the early diagnosis of candidemia.

## Background

Candidemia is a major infectious and steadily increasing complication of seriously immunocompromised patients that is associated with high mortality rates [1-5]. Non-specific clinical presentation, low positivity of blood cultures even in autopsy-proven cases and rapid course of the disease have necessitated the need for developing sensitive methods for the early diagnosis of invasive candidiasis [1,6]. Among the different approaches that have been developed and evaluated in the recent years include detection of *Candida *mannan [7,8], arabinitol [9-11], and nucleic acids [12-15]. However, all these methods have limitations of sensitivity and/or specificity. The recent introduction of BDG detection assay, a fungus-specific marker, has provided a new diagnostic tool with encouraging results [16-18]. This has encouraged the investigators to evaluate the usefulness of BDG in tandem of mannan and/or DNA for the early and specific diagnosis of invasive mycoses [19]. In this communication, we have evaluated the diagnostic value of *Candida *species-specific DNA, BDG, *Candida *mannan, and *Candida *anti-mannan antibodies in sera samples obtained from culture-proven candidemia patients and clinically suspected cases of candidiasis.

## Methods

### Patients and sera samples

Thirty-two sera from 27 culture-proven candidemia patients, 51 from 39 clinically suspected blood culture negative systemic candidiasis patients, 10 from *Candida *vaginitis patients and 16 from healthy subjects with no complaints of oral or vaginal *Candida *infection were included in the study. All the candidemia patients were admitted to the intensive-care unit and other wards of Mubarak Al-Kabeer Hospital, Kuwait for various conditions (Table 1). According to the European Organization for Research and Therapy of Cancer and Mycoses Study Group (EORTC/MSG) consensus revised definitions draft presented at ICAAC, 2005, all the patients yielding *Candida *species in blood cultures were considered as cases of candidemia. Besides yielding *Candida *species in blood culture, the patients had suggestive signs and symptoms of septicemia along with one or more risk factors, such as prolonged use of broad-spectrum antibiotics, presence of central venous catheter or extended period (> 2 weeks) of hospitalization. The other risk factors included gastrointestinal surgical procedures (n = 6), chronic renal failure (n = 3), multiple organ injury (n = 3), invasive urinary tract manipulation (n = 3), diabetes mellitus (n = 2) and pancytopenia (n = 2). In absence of microbiologic evidence of candidemia, 39 patients were included in the possible/suspected category of systemic candidiasis on the basis of suggestive host and clinical criteria. The patients included in this category had fever that did not respond to 4 days of broad-spectrum antibiotic therapy. In addition, they had at least three of the following risk factors: extended period of hospitalization (> 2 weeks), isolation of *Candida *species from one or more anatomic sites, presence of intravenous catheter/line, recent history of a surgical procedure, and administration of immunosuppressive therapy. Sera of healthy subjects were included as controls to derive baseline values. All the sera were stored at -20°C until used. The study was approved by the Committee for Protection of Human Subjects in Research, Faculty of Medicine. Kuwait University and informed written consent was obtained from the patients involved in the study.

**Table 1:** Particulars of 27 blood culture-positive candidemia patients and results on detection of species-specific Candida DNA, (1,3)-beta-D-glucan, mannan and anti-mannan antibodies by diagnostic kit cut-off value

Case no	Age/sex	Underlying condition	Blood culture	Semi-nested PCR with serum	BDG (pg/ml)	Mannan (ng/ml)	Anti-mannan Abs (AU/ml)
1	88/F	Septicemia, Chest infection	*C. albicans*	*C. albicans, C. parapsilosis*	35	0.32	9.8
1b*	88/F	Septicemia, Chest infection	*C albicans*	*C. albicans*	53	0.32	**11.2**
2	49/F	Chronic renal failure	*C. albicans*	*C. albicans*	40	0.26	0.8
3	79/M	Large bowel obstruction	*C. albicans*	*C. albicans*	**172**	**0.8**	**13.2**
4	17/M	Aseptic meningitis	*C. albicans*	*C. albicans*	20	0.28	2.4
5	34/M	Gut surgery	*C. albicans*	*C. albicans*	**130**	**0.66**	6.8
6	50/M	Pancytopenia	*C. albicans*	*C. albicans, C. parapsilosis*	30	**0.52**	0.8
7	56/M	Appendicular mass	*C. albicans*	*C. albicans*	44	**1.3**	**10.6**
7b*	56/M	Appendicular mass	*C albicans*	*C. parapsilosis*	69	0.38	9.6
8	35/M	-	*C. albicans*	*C. tropicalis*	**131**	**0.86**	**20.6**
9	42/M	-	*C. albicans*	*C. albicans*	43	**0.5**	3
10	26/F	Small bowel obstruction, pancreatitis	*C. albicans*	*C. albicans, C. parapsilosis*	32	0.48	8
10b*	26/F	Small bowel obstruction, pancreatitis	*C. albicans*	-ve	21	0.22	**34**
11	22/F	-	*C. albicans*	-ve	48	0.36	**24.4**
12	78/F	Laparotomy	*C. albicans*	-ve	28	0.3	**20.8**
12b*	78/F	Laparotomy	*C. albicans*	-ve	22	0.34	**27.8**
13	71/F	Laparotomy	*C. albicans*	*C. albicans, C. tropicalis*	**127**	**1.44**	2
14	9mo/F	Pneumonia	*C. albicans*	*C. parapsilosis*	**112**	**3.19**	1
15	68/M	Bladder cancer	*C. albicans*	*C. parapsilosis*	**157**	0.36	**35**
16	58/M	Renal failure	*C. albicans*	*C. albicans*	**254**	**1.54**	7
17	77/M	Chest trauma	*C. albicans*	*C. albicans*	**97**	**0.64**	1.6
18	48/M	Bronchopneumonia	*C. albicans*	*C. albicans*	**321**	0.24	**23.8**
19	20/F	Meningitis	*C. parapsilosis*	*C. albicans*	32	0.34	**11.2**
20	80/M	Urinary tract infection	*C. parapsilosis*	*C. parapsilosis*	**120**	0.3	**19.4**
21	54/F	Diabetes mellitus, LVF	*C. parapsilosis*	*C. parapsilosis*	65	0.22	1.4
22	04/M	Head injury	*C. parapsilosis*	*C. albicans*	20	0.28	2.2
23	6mo/F	Pneumonia	*C. parapsilosis*	*C. parapsilosis*	**101**	**0.62**	2.2
24	80/M	Chest infection	*C. tropicalis*	*C. tropicalis, C. albicans, C. parapsilosis*	47	0.48	7
25	41/M	Polyarteritis nodusa	*C. tropicalis*	*C. tropicalis*	**115**	**2.8**	1.4
25b*	41/M	Polyarteritis nodusa	*C. tropicalis*	*C. tropicalis*	**108**	0.4	**19.4**
26	74/M	Jaundice, fever	*C. tropicalis*	*C. parapsilosis*	**205**	**1.88**	**12.4**
27	28/M	Thrombocytopenia	*C. kruseii*	*C. albicans*	**132**	0.2	**13.4**

Age is expressed in years unless indicated. mo: months, LVF: left ventricular failure, BAL: broncho-alveolar lavage, M: male, F: female, * repeated specimen of the respective patient, – : not grown/information not available

Interpretation of results:

Mannan Ag: positive = > 0.5 ng/ml, intermediate = 0.25–0.5 ng/ml, negative = < 0.25 ng/ml; Anti-mannan antibodies: positive = > 10 AU/ml, intermediate = 5–10 AU/ml, negative = < 5 AU/ml; BDG: positive = = 80 pg/ml, equivocal = > 60 < 80 pg/ml, negative = < 60 pg/ml; positive results are shown in bold letters

### Candida species isolation and identification

Blood samples were processed for isolation of *Candida *species by BACTEC 9240 system (Becton Dickinson, Paramus, N.J. USA) using aerobic culture bottles. Aliquots from blood culture bottles yielding yeast growth were sub-cultured on Sabouraud dextrose agar plates with chloramphenicol (40 mg/L). A single representative colony was processed for identification by germ-tube test and Vitek 2 yeast identification system (bio Merieux Marcy l'Etoile, France). In case of discrepant results with snPCR identification, their identity was reconfirmed by ID 32C assimilation profile.

### Detection of Candida mannan

Mannan antigen was measured using a commercial sandwich immunoassay, Platelia *Candida *Ag (BioRad, Marnes La Coquette, France). The test was performed according to the instructions of the manufacturer. Briefly, each test serum (300 μl) was mixed with 100 μl of the treatment solution and placed in a boiling water bath for 3 minutes. After centrifugation, the supernatant was used for further testing. Fifty-μl of the conjugate and an equal amount of the treated serum supernatant was introduced into micro-titer plate wells pre-coated with anti-mannan monoclonal antibody. After incubation at 37°C for 90 min and 5 washing steps, 200 μl of the substrate buffer was added to each well, and the plates were incubated for 30 min at room temperature. The enzymatic reaction was terminated by adding the stopping solution and the optical density was read at 450 nm using a Tecan Spectra (Austria) plate reader. The reactions were performed in duplicates and each experiment included positive and negative controls as well as a calibration curve, which was made with a pool of normal human serum supplemented with known concentrations of mannan ranging from 0.1 to 2 ng/ml.

### Detection of Candida anti-mannan antibodies

Anti-mannan antibodies were measured using the Platelia *Candida *Ab/Ac/Ak kit, a two-stage indirect immunoenzymatic assay (Bio-Rad, Marnes La Coquette, France). The test was performed according to the manufacturer's instructions. In brief, 100 μl of each test serum diluted 1/6,400 was applied to each well of micro-titer plate wells sensitized with *C. albicans *cell wall mannan and the plate was incubated at 37°C for 1 h. After washing, 100 μl of the conjugate was added and the plate was incubated at 37°C for 1 h. After intensive washing, the reactions were revealed by 30 min of incubation in the dark with 200 μl of the substrate buffer. The enzymatic reaction was terminated by adding the stopping solution and the optical density was read at 450 nm using a Tecan Spectra plate reader. The reactions were performed in duplicates and each experiment included positive and negative controls as well as standard serum diluted to give four range points of 20, 10, 5, and 2.5 AU/ml for positive reference.

### (1,3)-beta-D-glucan assay

The test was carried out using the glucan detection kit, Fungitell (Associates of Cape Cod Inc., E. Falmouth, MA, USA). The glucan standard provided in the kit was mixed with reagent water to give a 100 pg/ml concentration. This was further diluted to obtain glucan concentrations of 50, 25, 12.5 and 6.25 pg/ml. Five μl of the serum sample was transferred to the designated wells of the micro-titer plate and 20 μl of the blood treatment reagent (0.6 M KCl and 0.125 M KOH) was added to each well containing the serum samples. The plate was shaken for 5 seconds and incubated for 10 minutes at 37°C. Twenty-five μl of each of the glucan standards (100, 50, 25, 12.5 and 6.25 pg/ml) was added to the designated wells. The provided Fungitell reagent was reconstituted with reagent water and pyrosol reconstitution buffer and 100 μl of this solution was added to each well. The plate was shaken for 5 seconds in a Tecan Sunrise (Austria) plate reader before reading at 405 nm every 1 minute for 40 minutes. The concentration of BDG in the clinical samples was calculated in comparison with a kinetic curve derived from known concentrations of glucan.

### PCR study

Reference *Candida *strains were obtained from American Type Culture Collection (ATCC, Manassas, Virginia, USA) and included *C. albicans *(ATCC 90029), *C. parapsilosis *(ATCC 10233), *C. tropicalis *(ATCC 750) and *C. glabrata *(ATCC 90030). All yeast strains were stored at -20°C in sterile distilled water. DNA was extracted from broth cultures by the method of Lee [20] with an additional step of DNA purification by extraction in phenol-chloroform (24:1). DNA from serum was extracted using the QIAamp DNA kit (QIAGEN) following the blood and body fluid spin protocol. All pan-fungal and species-specific forward and reverse primers as well as the DNA amplification method was the same as mentioned by Ahmad et al. [13]. To detect amplified DNA fragments, agarose gel electrophoresis was performed using 3% agarose gels as described previously [14]. The gels were exposed to UV light and photographed. The sizes of the amplified DNA fragments were identified by comparison with molecular size marker DNA (100-bp DNA ladder, Invitrogen).

### Interpretation of results and statistical analysis

The cut-off values recommended by the manufacturers for each test were used for determining positive, equivocal/intermediate and negative tests and were as follows: BDG, ≥ 80 pg/ml as positive, 60–79 pg/ml as equivocal and < 60 pg/ml as negative; mannan, > 0.5 ng/ml as positive, 0.25–0.5 ng/ml as intermediate and < 0.25 ng/ml as negative; anti-mannan *Candida *antibodies, > 10 AU/ml as positive, 5–10 AU/ml as intermediate and < 5 AU/ml as negative. All positive assay results were considered as true positives in patients with candidemia and also in clinically suspected patients. Additionally, all values under the cut-off values including doubtful results were considered as negative for calculation of sensitivity of the assays. Agreement between the qualitative test results was assessed by use of the kappa statistics. The p-values for the different tests were calculated using the Independent samples T test, Pearson's test and Z test for proportions using SPSS (Statistical package for social sciences) and Microstat.

## Results

### Detection and identification of Candida species by culture and snPCR

The particulars of 27 blood culture positive candidemia patients with respect to age, sex, underlying conditions, as well as results of detection of *Candida *DNA, BDG, and *Candida *mannan and anti-mannan antibodies are presented in Table 1. Eighteen were infected with *C*. *albicans*, 5 with *C. parapsilosis*, 3 with *C. tropicalis *and one with *C. krusei*. snPCR results were positive in 25 (92.5%) candidemia patients. Among 32 sera samples examined from 27 patients, 4 sera samples originating from three patients (Table 1, Case Nos. 10b, 11, 12 and 12b) were negative by snPCR. In 7 patients (Case Nos. 8,14, 15, 19, 22, 26, 27) *Candida *species isolated from blood cultures were identified differently by snPCR. The identity of *Candida *isolates showing discordant results was re-confirmed by Vitek2 yeast identification system and/or Chromagar *Candida*. In one patient (Case No. 7) while both the blood cultures yielded *C. albicans*, the second serum sample was snPCR positive for *C. parapsilosis*. In addition, snPCR detected 4 patients (Case Nos. 1, 6, 10, 13) with dual infection (3 with *C. albicans *and *C. parapsilosis *and one with *C. albicans *and *C. tropicalis*), and one patient (Case No. 24) was positive for *C. albicans*, *C. parapsilosis *and *C. tropicalis*.

### Candida mannan

Using a cut-off value of > 0.5 ng/ml for a positive mannan test, the sensitivity and specificity in candidemia patients were 41% and 100%, respectively. The quantities of mannan in positive serum samples ranged from 0.5 to 3.19 (mean ± SD = 1.29 ± 0.88) ng/ml) (Table 1).

### (1,3)-beta-D-glucan

Fourteen (52%) patients with proven candidemia were positive for BDG test using a cut-off value of 80 pg/ml. The amount of BDG in 15 positive serum samples ranged from 97 to 321 pg/ml (mean ± SD = 152.13 ± 63.08 pg/ml) (Table 1). The sensitivity and specificity of BDG test were 47% and 100%, respectively.

### Anti-mannan antibody

Using a cut-off value of > 10 AU/ml, the sensitivity of anti-mannan antibody test was 47% with 100% specificity. The antibody levels among the 15 positive sera samples from 14 patients ranged from 10.6 to 35 (mean ± SD = 19.81 ± 8.05) AU/ml. The 11 patients who yielded a negative test for mannan antigen were positive for anti-mannan antibodies (Table 1).

### Comparative analysis of Candida DNA, BDG, mannan and anti-mannan detection

**Figure 1:**
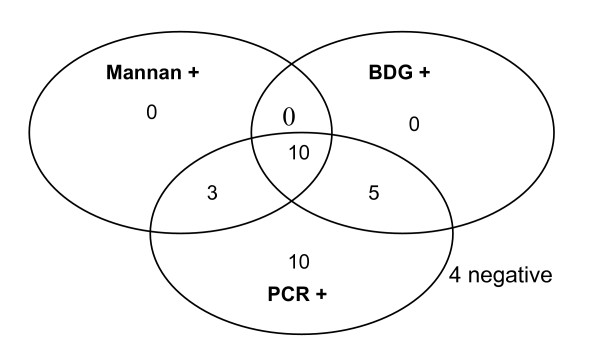
Venn diagram showing number of sera with positive results in each combination of mannan, BDG and snPCR (n = 32).

**Figure 2:**
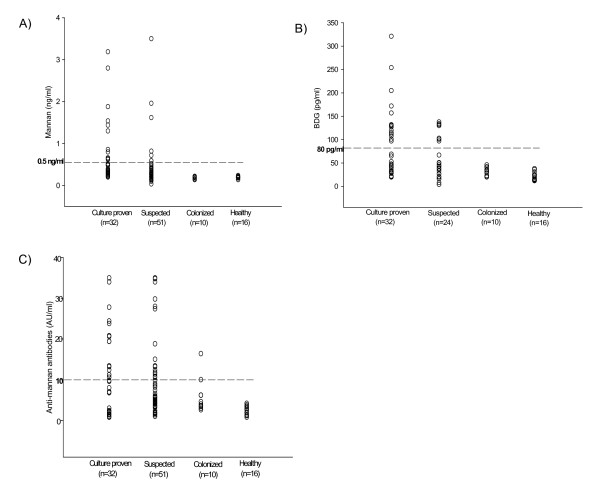
**Comparative results on the detection of *Candida *mannan (A), BDG (B) and Anti-mannan antibodies (C) in culture proven, clinically suspected and colonized patients and healthy controls**. The horizontal line in each plot represents the cut-off for a positive test.

**Figure 3:**
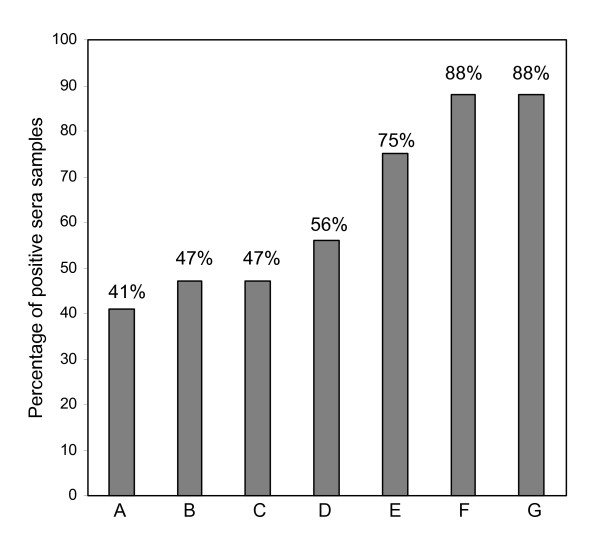
**Bar diagram showing the increasing sensitivity of the diagnostic tests to detect *Candida *infection in candidemia patients**. A: Mannan Ag, B: BDG, C: Anti-mannan Abs, D: Mannan + BDG, E: Mannan + Anti-mannan Abs, F: *Candida *DNA, G: Mannan + BDG + *Candida *DNA

**Figure 4:**
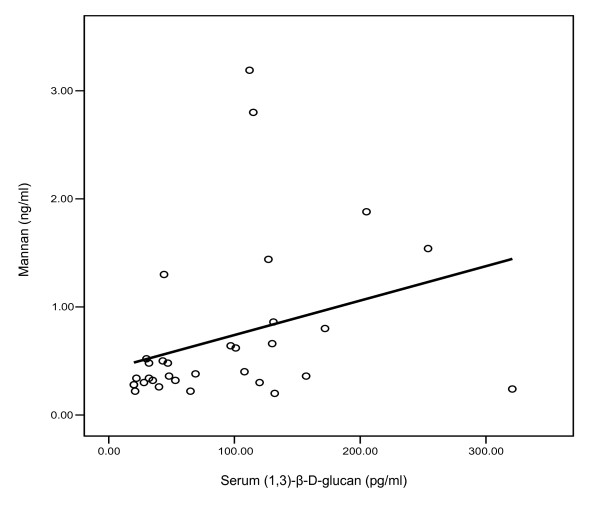
**Regression analysis of glucan and mannan values in culture-proven candidemia patients**. (n= 32, r^2 ^= 0.100, p = 0.078)

Using cut-off values recommended by the manufacturers, the comparative sensitivities of the assays for candidemia patients were as follows: *Candida *snPCR 88%, BDG 47%, mannan 41%, and anti-mannan antibodies 47% (Tables 1, 2 and Figures 1, 2, 3). Ten sera were positive for all the three markers that is, *Candida *DNA, BDG and *Candida *mannan (Figure 1) Fifteen sera samples positive for BDG were also positive by snPCR. None of the four sera samples that were negative by snPCR (Table 1; 10b, 11, 12, 12b), were positive for BDG or mannan, but all of them were positive for anti-mannan antibodies with values ranging from 20.8 -34 AU/ml (Table 1). Likewise, the 5 sera samples that were positive for BDG and negative for mannan were also uniformly positive for anti-mannan antibodies (Table 1; 15, 18, 20, 25b, 27). Only 4 of the 15 sera positive for anti-mannan antibodies were concomitantly positive for mannan. In general, increasing levels of BDG were associated with increasing levels of mannan. However, this correlation by Pearson test was not statistically significant (p = 0.078) (Figure 4). *Candida *DNA detection was found to be the most sensitive test (88%), followed by BDG and anti-mannan antibodies, 47% each, and mannan 41% (Figure 3). The combination of two tests improved the sensitivity for diagnosing invasive candidiasis/candidemia as follows: mannan and anti-mannan antibodies by ELISA 75%, mannan and BDG 56%, and mannan, BDG and *Candida *DNA 88% (Figure 3). The combined sensitivities for mannan and anti-mannan antibodies according to infecting *Candida *species were as: *C. albicans *(n = 22), 77%, *C. parapsilosis *(n = 5), 60% and *C. tropicalis *(n = 4), 75%, whereas for mannan and BDG, these were 55%, 40% and 75%, respectively (Table 2).

**Table 2:** Comparative results of mannan antigen, anti-mannan antibodies and (1,3)-beta-D-glucan alone and in combination in proven candidemia patients according to the infecting *Candida *species

*Candida *species in blood culture	No of sera tested	Number showing positive result by diagnostic kit value cut-off (%)				
		
		Mannan Ag	Anti-mannan Abs	BDG	Mannan + anti-mannan	Mannan + BDG
*C. albicans*	22	10 (45)	10 (45)	9 (41)	17 (77)	12 (55)
*C. parapsilosis*	5	1 (20)	2 (40)	2 (40)	3 (60)	2 (40)
*C. tropicalis*	4	2 (50)	2 (50)	3 (75)	3 (75)	3 (75)

### Clinically suspected candidemia patients

**Figure 5:**
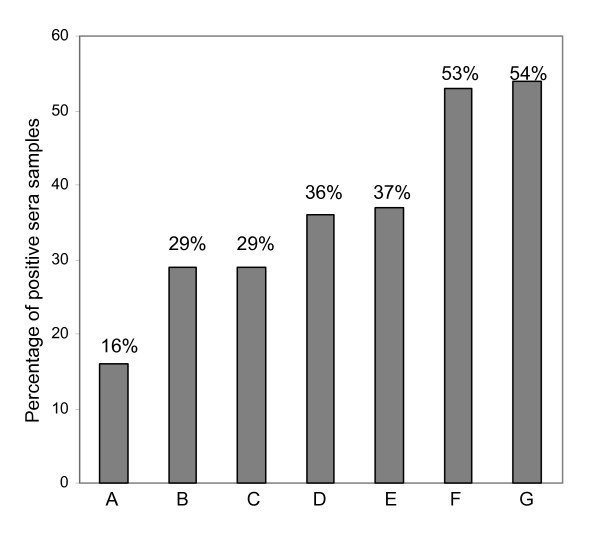
**Bar diagram showing the increasing sensitivity of the diagnostic tests to detect *Candida *infection in clinically suspected candidiasis patients**. A: Mannan Ag, B: BDG, C: Anti-mannan Abs, D: Mannan + BDG, E: Mannan + Anti-mannan Abs, F: *Candida *DNA, G: Mannan + BDG + *Candida *DNA

In the clinically suspected, blood culture negative category, 51 serum samples from 39 patients were tested. The positivities of the tests were as follows: *Candida *DNA was positive in 53%, BDG in 29%, mannan in 16%, and anti-mannan antibodies in 29% (Fig. 5). The combined detection of mannan and BDG, and mannan, BDG and *Candida *DNA enhanced the positivity to 36% and 54%, respectively (Figure 5). Serum samples of 10 patients were tested twice and of one patient thrice at different time intervals. In addition to *C. albicans*, 2 serum samples were positive for *C. tropicalis *and one for *C. parapsilosis *DNA. Of the 27 snPCR positive sera, 8 were also positive for anti-mannan, 5 for mannan and 6 for BDG (performed on 24 sera samples).

### Candida vaginitis patients and healthy controls

Sera from *Candida *vaginitis patients were uniformly negative for *Candida *DNA, BDG, and mannan (Figure 2). Sera of two patients showed positive values for anti-mannan antibodies (10 and 16.2 AU/ml) (Figure 2). None of the test markers were positive in sera of 16 healthy controls (Figure 2).

## Discussion

Diagnosis of invasive infections due to *Candida *species presents unique problems. Clinical and radiological signs are non-specific or develop late in the course of the disease. Conventional diagnostic tests are insensitive and the "gold standard" diagnostic procedures (histopathological examination and cultures from deep tissues) require aggressive approach, which is often not feasible due to thrombocytopenia, and the critical condition of these patients [21]. To overcome these limitations, assays for the detection of *Candida *antibodies, antigen, BDG and DNA have been developed and evaluated for the diagnosis of invasive candidiasis [8,16,22-28].

In the present study, we have retrospectively evaluated the diagnostic value of *Candida *DNA, *Candida *manan, and anti-mannan antibodies, and BDG individually and in comparison with each other in patients who yielded *Candida *species in blood cultures. snPCR has been successfully applied in the direct detection and species-specific identification of four clinically important *Candida *species (*C. albicans, C. parapsilosis, C. tropicalis *and *C. glabrata*) in sera samples. While species-specific *Candida *DNA was detected in 28 (88%) of the 32 sera samples obtained from 27 culture-proven candidemia patients, discordant results in comparison with Vitek2 identification were obtained in eight patients (Case Nos. 7b, 8, 14, 15, 19, 22, 26 and 27) (Table 1). This discrepancy in the results may be attributed to the possibility that these patients probably had concomitant infection with two different *Candida *species and only one of the infecting species was processed for identification by Vitek 2 method. Since we did not use a differential medium, such as Chromagar *Candida*, for making sub-cultures from BACTEC blood culture bottles, the possibility of missing one of the infecting species (probably with fewer colonies) existed. Four of the discordant results occurred between *C. albicans *and *C. parapsilosis *and one each between *C. tropicalis *and *C. parapsilosis *and *C. krusei *and *C. albicans*. Barring *C. krusei*, the other three *Candida *species were included in the snPCR protocol. Since some delay occurred between blood culture positivity and collection of serum samples, it is possible that detectable levels of the DNA of one of the two infecting *Candida *species were not available in the circulation when the blood was drawn for snPCR testing. This may also explain the reason as to why sera of four culture-positive candidemia patients (Cases Nos. 10b, 11, 12, 12b) were negative by snPCR. On the other hand, snPCR detected 5 candidemic patients whose all tests were negative except snPCR (Cases 2, 4, 10, 22, 24) and 5 additional patients (Case Nos. 1, 6, 10, 13 and 24) (Table 1), where more than one *Candida *species was involved besides *C. albicans*, and included *C. parapsilosis *in 4 and *C. tropicalis *in 2. Case No. 24 yielded positive results for *C. tropicalis *and *C. parapsilosis *besides *C. albicans*. These results support the previous reports that a reasonable proportion of patients with candidemia may have infection with more than one *Candida *species [13,29,30]. Since *Candida *species vary in their antifungal susceptibility profiles, this observation may be useful in administering appropriate therapy.

Recent studies have suggested that the combined detection of mannan and anti-mannan antibodies considerably improves the diagnosis of candidiasis [8,25,26]. While individual sensitivity of the test for mannan and anti-mannan antibodies in our study was only 41% and 47% respectively, the combined detection increased the sensitivity to 75% (Fig. 3). Sendid et al. [25] concluded that irrespective of the *Candida *species causing the disease, the combined sensitivity of mannan and anti-mannan antibody detection in candidiasis patients was > 80%. In our study, the combined sensitivities of mannan and anti-mannan antibodies for *C. albicans*, *C. parapsilosis*, and *C. tropicalis *were 77%, 60% and 75% and for mannan and BDG, these were 55%, 40% and 75%, respectively (Table 2). Additionally, there was also an inverse relationship between mannan and anti-mannan antibody levels, but it was not statistically significant (p = 0.063; data not shown) perhaps due to limited number of samples tested.

Some recent studies have demonstrated the usefulness of BDG estimation in the early diagnosis and management of fungal infections including candidiasis [16,19,31-33]. In our study, the sensitivity and specificity of BDG at a cut-off level of 80 pg/ml were 47% and 100%, respectively. The positive sera samples showed a range of 97 to 321 pg/ml (mean value 152.13 pg/ml). The sensitivity of BDG detection for diagnosing invasive fungal infections in different group of patients has been reported to vary considerably (50 to 100%), largely because of use of different cut-off values (10 to 120 pg/ml) for a positive test [16-19,31,34]. In a recent study, Pickering et al. [17] evaluated 39 sera samples from 15 patients with blood culture positive yeast infections using a cut-off value of 80 pg/ml. Thirty (77%) samples were positive for BDG (range 84 to 1359 pg/ml), and 13 of the 15 patients had at least one specimen positive. In a recent multi-center study of 107 patients with proven candidiasis, 81% had a positive result for BDG at a cut-off of 60 pg/ml and 78% had positive results at a cut-off of 80 pg/ml [18].

Our study is noteworthy in that it compared the diagnostic value of BDG in comparison with mannan using a quantitative EIA test in blood culture positive candidemia patients. The combination of the two tests improved the sensitivity to 56%. Our observation is in agreement with an earlier study by Mitsutake et al. [32]. These authors compared the specificities and sensitivities of enolase antigen, mannan antigen, Cand-Tec antigen and BDG in the diagnosis of 39 patients with candidemia. Using a cut-off value of 60 pg/ml, the specificity and sensitivity of BDG test were 84.4 and 87.5% respectively. The authors suggested that combination of two diagnostic assays may increase the accuracy of diagnosis of candidemia. Recently, Ostrosky-Zeichner et al. [18] have investigated the utility of the BDG assay in the diagnosis of fungal infections using a case control methodology. Using a cut-off value of 80 pg/ml, sensitivity and specificity of 64% and 92% was reported, respectively, with a PPV of 89% and NPV of 73%. However, in a subsequent analysis of the data of this study [18], Upton et al. [37] suggested that sensitivity and specificity of the test could heavily be influenced by prevalence rate of the disease in the patient population. Therefore, the calculation of PPV and NPV results from a population of selected case patients and unmatched control subjects may not provide useful information about the efficacy of the test. In an another study, Pazos et al. [19] reported that BDG and galactomannan exhibited similar *in vivo *kinetics in patients with invasive aspergillosis, hence their combined detection not only improved the specificity and PPV to 100% without affecting the sensitivity and NPV, but was also useful in identifying false positive reactions in each test. It seems that same may also be true for *Candida *mannan and BDG kinetics in patients with invasive candidiasis, hence their levels may increase or decrease in tandem. While increased levels of BDG in individual patients in our study were generally associated with increased levels of mannan, this correlation, however, was not significant by Pearson test (p = 0.078, r^2 ^linear = 0.1) (Figure 2). Nevertheless, the combined detection of mannan and BDG may also be helpful in eliminating false positive reactions which may occur in hemodialysis or ICU patients who may also have been colonized with *Candida *species [35,36]. In this context, all our *Candida *vaginitis patients were negative for BDG as well as *Candida *DNA and mannan. This is consistent with previous reports suggesting that *Candida *colonization may not lead to a positive BDG assay [16,19].

Our study has several limitations. Apart from the small numbers of samples tested, most of the observations are based on a single serum specimen obtained before initiating antifungal therapy. Additionally, there is no data available about the delay that occurred in obtaining a positive blood culture or between blood cultures and serum withdrawal for snPCR.

## Conclusion

The observations made in this study reinforce the diagnostic value of snPCR in the sensitive and specific diagnosis of candidemia. Additionally, in the absence of positive blood cultures, snPCR detected *Candida *DNA in sera of more than half of the clinically suspected patients. While detection of BDG, mannan and anti-mannan antibodies singly or in combination could help enhancing sensitivity and eliminating false positive tests, a more extensive evaluation of these assays in sequentially collected serum samples is required to assess their value in the early diagnosis of candidemia and also for monitoring response to antifungal therapy.

## Abbreviations

BDG = (1, 3)-β-D-glucan

PCR = Polymerase Chain Reaction

SnPCR = Seminested PCR

DNA = Deoxyribonucleic Acid

PPV = Positive Predictive Value

NPV = Negative Predictive Value

## Competing interests

The author(s) declare that they have no competing interests.

## Authors' contributions

ZUK and ASM conceived the study, supervised it and drafted the manuscript. FFA did the work which formed part of her Master thesis and contributed to writing of the manuscript. All authors have read and approved the final manuscript.

## Pre-publication history

The pre-publication history for this paper can be accessed here:


